# Risk Factors for Glaucoma Suspicion in Healthy Young Asian and Caucasian Americans

**DOI:** 10.1155/2014/726760

**Published:** 2014-07-21

**Authors:** E. Lauren Doss, Linden Doss, Ying Han, Susan Huang, Travis Porco, Melike Pekmezci, Shan Lin

**Affiliations:** ^1^Department of Ophthalmology, University of California, 10 Koret Way, San Francisco, CA 94143-0730, USA; ^2^School of Medicine, Loma Linda University, Anderson Street, Loma Linda, CA 92354, USA; ^3^Department of Epidemiology and Biostatistics, Division of Preventive Medicine and Public Health, 185 Berry Street, Lobby 5, Suite 5700, San Francisco, CA 94107, USA; ^4^Department of Pathology and Laboratory Medicine, University of California, Parnassus Avenue S512, San Francisco, CA 94143, USA

## Abstract

*Purpose.* To determine the prevalence of certain risk factors for glaucoma in a healthy, young population and to compare these risk factors between Asian Americans and Caucasians.* Methods.* 120 healthy graduate students (mean age 24.8 ± 3.0 years) underwent a comprehensive ophthalmic examination. Regression analyses controlling for age, sex, and refraction, comparing glaucoma risk factors in Asians (*n* = 54) and Caucasians (*n* = 41), were performed. Outcome variables included family history, intraocular pressure (IOP), spherical equivalent, central corneal thickness (CCT), mean deviation (MD) and pattern standard deviation (PSD), and disc and retinal nerve fiber layer (RNFL) parameters.* Results.* 61% of subjects were female; the mean spherical equivalent was −3.81 ± 3.2 D; and the mean axial length (AL) was 25.1 ± 1.7 mm. Regression analysis showed race affected spherical equivalent (*P* < 0.001), AL (*P* = 0.0073), IOP (*P* < 0.001), and cup to disc area ratio (CDAR) (*P* = 0.012). Family history, CCT, MD, and PSD did not vary between Asians and Caucasians (*P* > 0.05). In this study, we found Asian Americans, compared to Caucasians, had 2.95 ± 0.64 D greater myopia; greater IOP by 2.74 ± 0.62 mmHg; and larger CDAR by 0.12 ± 0.046.* Conclusions.* In our study population, young, healthy Asian Americans had greater myopia, IOP, and CDAR as compared to Caucasians, suggesting that racial variations can be important when diagnosing glaucoma.

## 1. Introduction

Glaucoma is a leading cause of irreversible blindness worldwide with significant prevalence in Asians [1–3]. The disease is characterized by gradual loss of retinal ganglion cells leading to thinning of the retinal nerve fiber layer (RNFL) and subsequent increase in the cup to disc ratio [4]. An increased risk of glaucoma may be indicated by a host of ocular biometrics including optic nerve parameters [4], central corneal thickness (CCT) [5], intraocular pressure (IOP) [6–8], and family history of glaucoma [8, 9].

According to the 2010 United States Census, the Asian population is the fastest growing of any other race group in the United States [10]. Glaucoma is the most common cause of permanent vision loss in Asian Americans [11]. A better understanding of the glaucoma risk factors in this population would promote greater public and medical awareness for glaucoma prevention and aid in its early recognition.

Although racial differences in the prevalence of glaucoma are documented [11], differences in the prevalence of risk factors leading to the development of glaucoma in young healthy populations have not been well studied. Recognizing these differences is essential not only in the early diagnosis of glaucoma but also in therapeutic decision making.

Several studies in Asia have examined individual glaucoma risk factors in patients with glaucoma or ocular hypertension [5–9, 12–18]. However, the confounding effects of socioeconomic and geographic factors in these various populations limit both the comparative power of  the findings and their applicability to Asian Americans. Other studies have analyzed individual risk factors in Asian Americans [19–21] or are in the process of collecting data on ocular biometrics and other risk factors in older Asian Americans [22]. In this study, we analyzed the complete risk factor profile associated with glaucoma in a unique cohort of healthy, young Asian Americans and Caucasian Americans.

## 2. Methods

One hundred twenty right eyes of 120 healthy graduate students between ages 21 and 40 years (mean 24.8 ± 3.0 years) from the University of California, San Francisco (UCSF), Schools of Dentistry, Medicine, and Pharmacy were enrolled in this cross-sectional study. Study participants were recruited in 2009 by email to the class list serves. Examinations were performed at the Beckman Vision Center of  UCSF by a single board-certified ophthalmologist (SL). Written informed consent was obtained from each participant. The Committee on Human Research at UCSF approved this study prior to data collection.

Participants responded to a brief questionnaire that included questions about past medical history, family history of glaucoma, history of laser in situ keratomileusis (LASIK), and ethnic self-identity. Comprehensive ophthalmic examination included visual acuity, refraction, indirect ophthalmoscopy, IOP measured by Goldmann tonometry, automated perimetry, and optical coherence tomography (OCT) to evaluate both the anterior segment and the optic nerve. The Swedish interactive threshold algorithm (SITA) 24-2 protocol on the Humphrey Field Analyzer (HFA2, Carl Zeiss Meditec, Inc., Dublin, CA) was used for standard automated perimetry. Axial length measurements were obtained by IOLMaster (Carl Zeiss Meditec, Inc., Dublin, CA); central corneal thickness and anterior chamber depth were measured using the anterior segment optical coherence tomograph (Visante OCT, Carl Zeiss Meditec, Inc., Dublin, CA). Disc parameters and RNFL were assessed using Fourier-domain optical coherence tomography (FD-OCT, RTVue-100, Optovue, Fremont, CA).

Inclusion criteria included (1) age 18 years or older, (2) self-declared white or Asian ancestry (Chinese, Filipino, East Indian, Korean, Vietnamese, and Others), (3) enrollment in School of Medicine, Dentistry, or Pharmacy at UCSF as a first year student, and (4) ability to perform all required testing as described above. Exclusion criteria included (1) IOP > 21 mmHg, (2) significant ocular disease, (3) history of intracranial disease or intraocular surgery, and (4) presence of systemic disease which could affect optic disc configuration such as diabetes mellitus or severe hypertension.

### 2.1. FD-OCT

**Figure 1:**
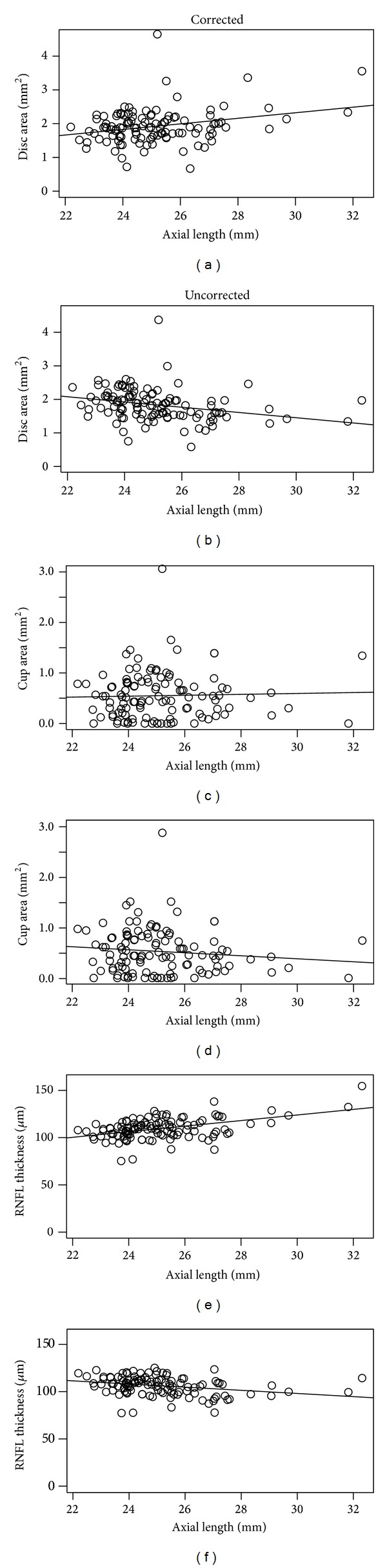
Comparison of posterior segment optical coherence tomography results, corrected and uncorrected for axial length [23]. RNFL: nerve fiber layer.

Optovue RTVue-100 OCT (software version 2.0.4.0) imaging was obtained in each subject without dilation by a single, experienced examiner. Optic nerve head analysis was performed with the three-dimensional disc and nerve head map 4-mm diameter (NHM4) RTVue protocols. The automated determination of the disc margin as delineated by the edge of the retinal pigment epithelium was used in calculating optic disc measurements in this study. RNFL analysis was performed using the fast RNFL algorithm (version 3.4) provided with the RTVue OCT instrument. Optic nerve measurements by OCT included disc area, cup area, rim area, rim volume, cup volume, cup to disc area ratio (CDAR), horizontal cup to disc ratio (CDR), vertical CDR, RNFL thickness, superior hemisphere RNFL thickness, and inferior hemisphere RNFL thickness. Variables were corrected for the influence of axial length by factor (3.382)^*d*^(0.01306)^*d*^(*x* − 1.82)^*d*^, where *d* is the dimension, that is, 1 for linear measurements, 2 for area measurements, and 3 for volumes, and *x* = AL as previously published (Figure 1) [23].

### 2.2. Statistical Analysis

Linear regression was conducted for continuous variables; logistic regression was conducted for binary outcomes. We conducted analyses controlling for age, sex, and refraction, comparing glaucoma risk factors in Asians (*n* = 54) and Caucasians (*n* = 41). Measured variables included family history, IOP, spherical equivalent, axial length (AL), central corneal thickness (CCT), mean deviation (MD), pattern standard deviation (PSD), and disc and RNFL parameters. We included only right eyes in the analysis. All tests were 2-sided, and a *P* value less than 0.05 was considered statistically significant. Statistical analyses were performed using R version 2.12 for Macintosh (R Foundation for Statistical Computing, Vienna, Austria).

## 3. Results

One hundred twenty right eyes of young, healthy subjects who met the inclusion criteria were analyzed in this study. Subjects had a mean age of 24.8 ± 3.0 years, mean spherical equivalent of –3.81 ± 3.19 D, and mean axial length (AL) of 25.1 ± 0.04 mm. Seventy-three (61%) were female. Fifty-four self-identified as Asian (35 Chinese, 4 Filipino, 5 East Indian, 5 Korean, 5 Vietnamese) and 41 as Caucasian (Table 1).

When myopia was examined as a potential confounder in OCT measurements, as expected, uncorrected parameters measured by OCT exhibited a strong trend toward decreasing size as myopia increased. This described phenomenon can be eliminated through factoring in axial length (Figure 1) [23].

Regression analysis controlling for age, sex, and refractive error showed that Asian American ethnicity was significantly associated with lesser spherical equivalent (greater myopia; *P* < 0.001), longer axial length (*P* = 0.007), greater IOP (*P* < 0.001), and greater cup to disc area ratio CDAR (*P* = 0.012) (Table 2). Other risk factors including thickness of the retinal nerve fiber layer, family history (OR 0.83; 95% CI: 0.29, 2.37), central corneal thickness, Humphrey mean deviation, and Humphrey pattern standard deviation did not vary significantly between Asians and Caucasians (*P* > 0.05). In this study, we found that on average Asian Americans, compared to Caucasians, had 2.95 ± 0.64 D greater myopia; their average IOP was greater by 2.74 ± 0.62 mmHg; and cup to disc area ratio was 0.12 ± 0.046 larger.

## 4. Discussion

Our results indicate that in a selected population of young, healthy graduate students, Asian American ethnicity, independent of age or sex, is associated with an oculometric profile marked by greater IOP, increased myopia, and larger CDAR compared to Caucasian American ethnicity. However, family history of glaucoma, RNFL, and CCT did not vary significantly between these ethnic groups.

The extent to which risk factors for development of glaucoma in individuals with healthy eyes are the same as those for its progression is not completely clear; however, the risk factors and biometric predictors examined in this study were chosen for their statistical associations and clinical importance. For the same reasons, eye care providers tend to assume their causal association with the development of glaucoma [24]. In this study, we looked at some of these risk factors strongly supported by evidence and compared between Asian Americans and Caucasian Americans. These putative factors include elevated IOP [6–8], increased cup to disc ratio [8], CCT [5], refractive error [12–17], and family history of glaucoma [8, 9].

We found IOP to be greater by 2.74 ± 0.62 mmHg in Asian Americans compared to Caucasian Americans in healthy, young students. IOP as a risk factor for the development of primary open angle glaucoma (POAG) has been investigated in different populations [6–8]. In these studies, there was a 10% to 14% increased risk of developing glaucoma over the following 5 to 9 years in subjects with baseline IOPs 1 mmHg or greater than average. Previous studies have concluded that CCT-adjusted IOP is higher in the African American community (16.12 ± 3.27 mmHg) than the Caucasian community (14.32 ± 2.93 mmHg), but not in the Asian American community [21, 25]. However, we found that healthy, young Asian Americans have higher IOP than Caucasians. This may be due to the subjects' age difference between our study and other studies, wherein more elderly subjects were included. In addition, hypertension, body mass index, and other lifestyle indicators that are more prominent in the elderly than the young population affect IOP [8, 26].

In our Asian American cohort, vertical and horizontal cup ratios were larger, and the CDAR (controlled for refractive error) was greater by 0.12 ± 0.046 compared to Caucasians, consistent with our group's past findings in these ethnic groups [19]. While the CDAR has not been studied as extensively as the cup to disc ratio, it is of note that a cup to disc ratio greater than 0.7 has been associated with an increased risk of glaucoma [8], and in some populations, risk of development of POAG is increased by 25% for each increase of 0.1 in horizontal cup to disc ratio and by 32% for the same incremental increase in vertical cup to disc ratio [27]. It is unlikely that the larger cup size and greater CDAR in these young Asian Americans necessarily denote glaucoma suspicion or disease, because our subjects have normal visual fields and nerve fiber layer analyses.

Myopia has long been identified as a risk factor for POAG [12–16] associated with a 2- to 3-time higher risk of glaucoma [27]. Myopia and glaucoma are increasingly prevalent in Asian populations [28]. In this study we found that greater myopia (2.9 D) is more common in young Asian Americans than in Caucasians within our cohort. Morphologic optic nerve head changes often associated with myopia can mimic or mask glaucomatous changes complicating diagnosis and monitoring [29–31]. Optic disc tilting, associated with myopia, is present in about four out of 1000 eyes of adult Chinese in Northern China [32]. Doshi et al. identified a small cohort of young Chinese-American males erroneously diagnosed with glaucoma or considered glaucoma suspects, but who had stable ocular findings, and attribute this condition to myopia and tilted discs [31]. Many were being treated with IOP-lowering therapy for glaucoma, a condition they may not have had.

CCT measurement, a significant predictor of higher risk of developing severe glaucomatous change, [5] has become routine in glaucoma management yet does not vary significantly between Asian and Caucasian-American glaucoma patients, [21] consistent with our findings. Interestingly we also found no racial variation in self-reported heritance patterns, although familial glaucoma history has been associated with the presence and severity of POAG in Chinese [9].

The limitations of our study bear mentioning. Ideally all data would be derived from large prospective population-based cohorts, particularly given the definition of glaucoma as a progressive disease, associated with environmental factors such as minimal educational attainment [33] and urban locale [34–37]. Along these lines, our sample sizes for individual Asian racial subgroups were inadequate to support a subgroup analysis. We hope that our results will encourage further investigation into the variation among these ethnic subgroups. As with all studies in which multiple comparisons are performed, chance could play a role in some of the significant associations observed. To avoid this risk, we have deliberately chosen all relevant potential risk factors based on previous studies. Although this study may be influenced by a selection bias inherent in the voluntary recruitment process, we minimized the possibility of this and other biases through controlled analyses for known confounding variables and minimized interobserver and interoperative variation through standardization of the examiner and clinic setting. It is of note that we did not include gonioscopy in this study due to the poor tolerance of this healthy volunteer population to the relatively invasive gonioscopic examination. Instead we opted to minimize participant discomfort by using anterior segment tomography as a way to assess the angles. We found no narrow angles, which is not surprising given the young age of our cohort. Lastly, when comparing our study with others, it is imperative to keep in mind the young age and other unique demographics of our cohort subjects, being Asian American graduate students, compared to most other studies on the subject. While we believe this makes our study particularly intriguing and innovative, we advise caution when generalizing these results.

Our findings suggest that Asian Americans tend to have higher IOP, higher myopia, and greater cup to disc ratio making them appear more suspicious for glaucoma than Caucasians in a healthy young population. According to these findings, Asian Americans may be at higher risk of developing glaucoma, making it imperative to bear in mind these racial variations when diagnosing glaucoma. To determine whether these positive parameters confer increased risk of the disease requires further study. We hope that this study will serve as a starting point for the longitudinal evaluation of glaucoma risk factors in this growing population.

## Figures and Tables

**Table 1:** Demographic and ocular characteristics (mean ± standard deviation) for all subjects, the Asian American subgroup, and Caucasian American subgroup. RNFL: retinal nerve fiber layer. All optical coherence tomography (OCT) measurements were taken with the RTVue-100 Fourier-domain OCT.

	All subjects	Asian Americans	Caucasian Americans
Variable (mean ± SD)	*n* = 120	*n* = 54	*n* = 41
Age in years	24.83 ± 2.99	24.35 ± 3.02	25.48 ± 2.36
Female	73/120 (61%)	33/54 (61%)	30/41 (72%)
Spherical equivalent (D)	−3.81 ± 3.19	−5.57 ± 3.38	−2.67 ± 2.23
IOP (mmHg)	13.83 ± 3.05	15.24 ± 3.2	12.65 ± 2.3
Disc area (mm)	1.92 ± 0.52	2.00 ± 0.54	1.84 ± 0.50
Cup area (mm)	0.55 ± 0.47	0.63 ± 0.50	0.45 ± 0.38
Rim area (mm)	1.37 ± 0.42	1.37 ± 0.42	1.40 ± 0.41
Rim volume (mm^3^)	0.21 ± 0.14	0.21 ± 0.15	0.23 ± 0.13
Cup volume (mm^3^)	0.10 ± 0.14	0.13 ± 0.18	0.06 ± 0.08
Cup to disc area ratio	0.27 ± 0.19	0.30 ± 0.18	0.23 ± 0.18
Horizontal cup to disc ratio	0.55 ± 0.25	0.62 ± 0.19	0.46 ± 0.28
Vertical cup to disc ratio	0.47 ± 0.23	0.51 ± 0.20	0.42 ± 0.26
RNFL thickness (*μ*m)	109.49 ± 11.1	112.51 ± 9.46	107.13 ± 12.8
Superior hemisphere thickness (*μ*m)	106.98 ± 16.2	111.67 ± 10.7	104.75 ± 14.5
Inferior hemisphere thickness (*μ*m)	111.10 ± 11.5	113.35 ± 10.2	109.56 ± 13.1
Central corneal thickness (*μ*m)	530 ± 40	530 ± 40	540 ± 40
Axial length (mm)	25.1 ± 0.04	25.81 ± 0.64	24.62 ± 1.66
Anterior chamber depth (mm)	3.34 ± 0.25	3.33 ± 0.28	3.36 ± 0.22

**Table 2:** Regression analysis comparing several variables between Asian Americans and Caucasian Americans, controlling for age and sex. Axial length was included in the adjustment for those variables marked with an asterisk (∗). SE: standard error. Measurements were taken with Fourier-domain optical coherence tomography.

Variable	Effect size (Asians as reference) ± SE	*P* value
Spherical equivalent (D)	−2.95 ± 0.64	<0.001
Axial length (mm)	−1.03 ± 0.38	0.007
Intraocular pressure (mmHg)	−2.74 ± 0.62	<0.001
Retinal nerve fiber layer thickness (*μ*m)∗	−3.60 ± 2.73	0.19
Cup to disc area ratio∗	−0.12 ± 0.046	0.012
Central corneal thickness (*μ*m)	−1.66 ± 8.20	0.84
